# Spinal Muscular Atrophy Patient iPSC-Derived Motor Neurons Have Reduced Expression of Proteins Important in Neuronal Development

**DOI:** 10.3389/fncel.2015.00506

**Published:** 2016-01-11

**Authors:** Heidi R. Fuller, Berhan Mandefro, Sally L. Shirran, Andrew R. Gross, Anjoscha S. Kaus, Catherine H. Botting, Glenn E. Morris, Dhruv Sareen

**Affiliations:** ^1^Wolfson Centre for Inherited Neuromuscular Disease, The Robert Jones and Agnes Hunt Orthopaedic HospitalOswestry, UK; ^2^Institute for Science and Technology in Medicine, Keele UniversityStaffordshire, UK; ^3^Board of Governors-Regenerative Medicine Institute, Cedars-Sinai Medical CenterLos Angeles, CA, USA; ^4^iPSC Core, The David and Janet Polak Foundation Stem Cell Core LaboratoryLos Angeles, CA, USA; ^5^BSRC Mass Spectrometry and Proteomics Facility, University of St AndrewsFife, UK; ^6^Department of Biomedical Sciences, Cedars-Sinai Medical CenterLos Angeles, CA, USA

**Keywords:** SMA, UBA1, UCHL1, Proteomics, Human iPSCs, Neuronal Development, Motor neuron

## Abstract

Spinal muscular atrophy (SMA) is an inherited neuromuscular disease primarily characterized by degeneration of spinal motor neurons, and caused by reduced levels of the SMN protein. Previous studies to understand the proteomic consequences of reduced SMN have mostly utilized patient fibroblasts and animal models. We have derived human motor neurons from type I SMA and healthy controls by creating their induced pluripotent stem cells (iPSCs). Quantitative mass spectrometry of these cells revealed increased expression of 63 proteins in control motor neurons compared to respective fibroblasts, whereas 30 proteins were increased in SMA motor neurons vs. their fibroblasts. Notably, UBA1 was significantly decreased in SMA motor neurons, supporting evidence for ubiquitin pathway defects. Subcellular distribution of UBA1 was predominantly cytoplasmic in SMA motor neurons in contrast to nuclear in control motor neurons; suggestive of neurodevelopmental abnormalities. Many of the proteins that were decreased in SMA motor neurons, including beta III-tubulin and UCHL1, were associated with neurodevelopment and differentiation. These neuron-specific consequences of SMN depletion were not evident in fibroblasts, highlighting the importance of iPSC technology. The proteomic profiles identified here provide a useful resource to explore the molecular consequences of reduced SMN in motor neurons, and for the identification of novel biomarker and therapeutic targets for SMA.

## Introduction

Spinal Muscular Atrophy (SMA) is a recessively inherited neuromuscular disease displaying a wide range of severity, from the most severe Type I (diagnosed somewhere between birth to 6 months of age), to adult onset, Type IV. SMA is primarily characterized by loss of function and degeneration of lower motor neurons in the anterior horn of the spinal cord, and is caused by reduced levels of the survival of motor neurons (SMN) protein, which is encoded by two genes, *SMN1* and *SMN2* (Lefebvre et al., 1995). Most of the mRNA transcribed from the *SMN2* gene is alternatively spliced to omit exon 7 and any protein translated from such “delta7” mRNA is unstable and rapidly degraded (Lefebvre et al., 1995; Lorson et al., 1998; Pellizzoni et al., 1999). In SMA patients, the *SMN1* gene is mutated or deleted and only a small amount of stable and functional SMN is produced from the *SMN2* gene, with the more severe phenotypes having the least SMN (Coovert et al., 1997; Lefebvre et al., 1997).

SMN is a ubiquitously-expressed protein that plays a central role RNA biogenesis; regulating the assembly of small nuclear ribonucleic proteins (snRNPs) in the cytoplasm and their subsequent transport into the nucleus (Lefebvre et al., 1995; Pellizzoni et al., 1998). Aside from this housekeeping role, SMN also appears to have a neuronal-specific role in mRNA processing, where it interacts with hnRNP-R to transport β-actin mRNA in axons (Rossoll et al., 2003; Carrel et al., 2006). Despite this knowledge about the cellular functions of SMN, it has become clear, from studies with mouse models, that defects in RNA splicing or axonal transport do not fully explain why lower motor neurons are particularly vulnerable to reduced levels of SMN (Kariya et al., 2008; Burghes and Beattie, 2009; Murray et al., 2010; Sleigh et al., 2011; Hamilton and Gillingwater, 2013).

Previous attempts to understand the molecular consequences of reduced SMN expression in SMA have largely been focused on patient fibroblasts and animal models. Various animal models of SMA are available (Edens et al., 2015), but their intrinsic differences from humans may prevent effective translation to clinical trials. In addition, animal models of SMA may not be as amenable to high-throughput drug discovery programs, compared to patient cells. SMA patient skin fibroblasts are easily accessible and can be expanded in culture, in large quantities, with relative ease. Although, SMA patient skin fibroblasts display reduced SMN levels in culture, the skin itself is pathophysiologically spared in patients, suggesting that these cells respond differently to, or have different requirements for, SMN, compared with lower motor neurons.

Reprogramming somatic cell types to pluripotency by human induced pluripotent stem cell (iPSC) technology preserves the patient's genome and its errors and allows investigators to observe these diseased genotypes within any human cell type (Takahashi et al., 2007; Yu et al., 2007). Human iPSCs can provide an unlimited supply of patient cells (e.g., lower motor neurons for SMA), which can then be studied *in vitro*. We have previously shown that iPSCs from type I SMA patients are capable of differentiating into motor neurons that lack *SMN1* expression and demonstrate selective motor neuron death over time (Ebert and Svendsen, 2010; Sareen et al., 2012; Barrett et al., 2014). Whilst targeted biochemical studies enable the characterization of known protein pathways in such cellular models, large-scale quantitative mass spectrometry approaches offer the possibility of studying the proteome in an unbiased fashion, and can be useful for assessing the suitability of cellular models (Hornburg et al., 2014).

The aim of this study was to conduct the first comprehensive evaluation of the proteome of SMA patient iPSC-derived motor neurons and provide a comparison against genetically matched fibroblasts using quantitative mass spectrometry (i.e., iTRAQ). We were particularly interested to examine whether there are down-stream effects of reduced SMN in iPSC-derived motor neuron cultures, not found in fibroblasts, as these could be useful for exploring the particular vulnerability of motor neurons in SMA. In a 4-plex quantitative proteomics comparison (iTRAQ), we compared the proteome of SMA and control motor neurons with the fibroblast cell lines from which they were originally derived. We provide evidence that motor neurons from SMA patients display reduced expression of proteins involved in developmental and differentiation pathways, including ubiquitin-activating enzyme 1 (UBA1) and ubiquitin carboxyl-terminal esterase L1 (UCHL1), and that most of these changes are distinct from those seen in the fibroblast cell lines from which the iPSCs were derived.

## Methods

### Ethics statement

Human dermal fibroblasts or lymphoblastoid cell lines (LCLs) were obtained from the Coriell Institute for Medical Research. The Coriell Cell Repository maintains the consent and privacy of the donor LCLs. All the cell lines and protocols in the present study were carried out in accordance with the guidelines approved by Stem Cell Research Oversight committee (SCRO) and Institutional Review Board (IRB) at the Cedars-Sinai Medical Center under the auspice IRB-SCRO Protocols Pro00032834 (iPSC Core Repository and Stem Cell Program), Pro00024839 (Using iPS cells to develop novel tools for the treatment of SMA) and Pro00036896 (Sareen Stem Cell Program).

### Generation of human iPSCs using episomal plasmids

Human iPSCs were generated as described previously (Sareen et al., 2012, 2013, 2014; Barrett et al., 2014). Briefly, iPS cell lines were reprogrammed from dermal fibroblasts into virus-free iPSC lines with the Nucleofector Kit using 1.5 μg of each episomal plasmid (Addgene) expressing six factors: OCT4, SOX2, KLF4, L-MYC, LIN28, and p53 shRNA (pCXLE-hOCT3/4-shp53-F, pCXLE-hUL, and pCXLE-hSK). This method has a significant advantage over viral transduction because exogenously introduced genes do not integrate and are instead expressed episomally in a transient fashion. Dermal fibroblasts (1 × 10^6^ cells per nucleofection) were harvested, centrifuged at 1500 rpm for 5 min, re-suspended carefully in Nucleofector® Solution and the U-023 program was applied. These nucleofected cells were plated on feeder-independent BD Matrigel™ growth factor-reduced Matrix (Corning/BD Biosciences, #354230). All cultures were maintained at 20% O2 during the reprogramming process. Individual iPSC colonies with ES/iPSC-like morphology appeared between day 25 and 32 and those with best morphology were mechanically isolated, transferred onto 12-well plates with fresh Matrigel™ Matrix, and maintained in mTeSR®1 medium. The iPSC clones were further expanded and scaled up for further analysis.

### Karyotype

The SMA and control iPS cell lines were incubated in Colcemid (100 ng/mL; Life Technologies) for 30 min at 37°C and then dissociated using trypsin (TrypLE) for 10 min. They were then washed in phosphate buffered saline (PBS) and incubated at 37°C in 5 mL of hypotonic solution (1 g potassium chloride (KCl), 1 g sodium citrate in 400 mL water) for 30 min. The cells were centrifuged for 2.5 min at 1500 rpm and resuspended in fixative (methanol: acetic acid, 3:1) at room temperature for 5 min. This was repeated twice, and finally cells were resuspended in 500 μL of fixative solution and submitted to the Cedars-Sinai Clinical Cytogenetics Core for G-Band karyotyping.

### Pluritest

High quality total RNA was isolated from undifferentiated iPSCs, H9 hESCs, fibroblasts, and primary human neural progenitors using the RNeasy Mini Kit (Qiagen) and subsequently run on a Human HT-12 v4 Expression BeadChip Kit (Illumina). The raw data file (idat file) was subsequently uploaded on to an open-source and easily accessible Pluritest widget online (www.pluritest.org). PluriTest is a transciptomics and bioinformatics based characterization test for determining pluripotency of a reprogrammed cell line (Müller et al., 2008–2012). In this test mRNA expression values of all probes including pluripotency-associated genes are scored against samples in the stem cell model matrix, consisting of 264 pluripotent cell lines (223 hESC and 41 human iPSC) and 204 samples derived from somatic cells and tissues. The red and blue background hint to the empirical distribution of the pluripotent (red) and non-pluripotent samples (blue) in the Müller et al. (2008–2012) test data set. An iPSC line is considered a bona-fide fully reprogrammed pluripotent cell line when the pluripotency score is above 20 and the novelty score is below 1.6. A typical chart combines pluripotency score on y-axis and novelty score on x-axis.

### Neural and motor neuron differentiation

The SMA patient and control subject iPSCs were grown until ~90% confluent as colonies under normal maintenance conditions before the start of the differentiation. The single cell iPSCs were gently lifted by accutase treatment for 5 min at 37°C. Subsequently, 1.5–2.5 × 10^4^ cells were placed in each well of a 384 well plate in defined neuroectodermal differentiation medium (NDM) composed of Iscove's modified Dulbecco's medium supplemented with B27–vitamin A (2%) and N2 (1%), with the addition of 0.2 μM LDN193189 and 10 M SB431542 (NDM+LS). This is a modified dual-SMAD protocol (Chambers et al., 2009). All days of differentiation described are post-iPSC (PI) stage (day 0). At day 2 PI, neural aggregates were transferred to low adherence polyhydroxyethylmethacrylate (poly-HEMA) coated flasks and cultured in suspension. After this point, the differentiation protocol was optimized to reliably generate lower spinal motor neurons. At 5 days PI, neuroectodermal aggregates were seeded on laminin-coated (50 μg/mL; Sigma #L2020) six well plates to induce neural rosette formation. From days 12 to 19 PI, the media was changed to motor neuron specification media (MNSM) supplemented with 0.25 μM all-trans retinoic acid (ATRA), 1 μM purmorphamine, 20 ng/mL brain-derived neurotrophic factor (BDNF), 20 ng/mL glial cell line-derived neurotrophic factor (GDNF), 200 ng/mL ascorbic acid, and 1 μM dibutyryl cyclic adenosine monophosphate (db-cAMP). Between days 17 and 19 PI, neural rosettes were selected using rosette selection media (StemCell Technologies, #05832). The isolated rosettes were subsequently cultured in motor neuron precursor expansion media (MNPEM) consisting of NDM, 0.1 μM ATRA, 1 μM purmorphamine, 100 ng/mL EGF, and 100 ng/mL FGF2. These iPSC-derived motor neuron precursor spheres (iMPS) can be expanded over a 2–7 week period by an automated chopping method (Svendsen et al., 1998). The iMPS were differentiated further for 21 and 28 days, for maturation into into motor neurons before harvesting or fixation. These motor neuron samples were used in iTRAQ and other experiments described here. Briefly, iMPS were dissociated with accutase and then seeded onto laminin-coated plates (5–8 × 10^5^ cells/cm^2^) in MN maturation media (MNMM) stage 1 for 7 days consisting of NDM, supplemented with 0.1 μM ATRA, 1 μM purmorphamine, 10 ng/mL BDNF, 10 ng/mL GDNF, 200 ng/mL ascorbic acid, 1 μM db-cAMP, and 2.5 μM *N*-[(3,5-Difluorophenyl)acetyl]-L-alanyl-2-phenyl]glycine-1,1-dimethylethyl ester (DAPT; inhibitor of γ-secretase; Cayman Chemicals, #13197). The remainder of terminal differentiation was carried out in Neurobasal supplemented with 1% NEAA, 0.5% GlutaMax, 1% N2, 10 ng/mL BDNF, 10 ng/mL GDNF, 200 ng/mL ascorbic acid, 1 μM db-cAMP, and 0.1 μM.

### Quantitative proteomics comparison

Cell pellets (containing ~6 × 10^4^ cells) were extracted in four volumes of extraction buffer (w/v) containing 6 M Urea, 2 M Thiourea, 2% CHAPS and 0.5% SDS in HPLC-grade water (Sigma Chromasolv plus). The extracts were sonicated briefly and left on ice for 10 min, followed by centrifugation at 13,000 × g for 10 min at 4°C to pellet any insoluble material. The proteins were precipitated in six volumes of ice cold acetone overnight at −20°C. The acetone precipitates were pelleted by centrifugation at 13,000 × g for 10 min at 4°C and the supernatant was carefully removed and discarded. The pellets were allowed to air-dry, and were then resuspended in 500 mM tetraethylammonium bromide (TEAB). The protein concentration in each group was determined using a Bradford assay.

### Sample preparation for mass spectrometry analysis

Reduction, alkylation, and digestion steps were performed according to the recommendations detailed in the iTRAQ labeling kit (AB Sciex). The extracts were digested with trypsin (5 μg per 100 μg of protein) overnight at 37°C, followed by reduction and alkylation steps performed according to instructions outlined in the iTRAQ labeling kit. The digests were subsequently dried down in a vacuum centrifuge and iTRAQ labeling was carried out according to the instructions in the iTRAQ labeling kit. The iTRAQ tags were assigned to samples as follows: 114, fibroblasts from control GMO3814 (*n* = 1); 115, motor neurons—derived from control GMO3814 (*n* = 1); 116, fibroblasts from Type 1 SMA patient GMO0232 (*n* = 1); 117, motor neurons—derived from Type 1 SMA patient GMO0232 (*n* = 1). Each tag was incubated with 60 μg of total protein (as determined by a Bradford protein assay).

### Mass spectrometry analysis

The combined 4-plex iTRAQ labeled peptides were concentrated in a vacuum concentrator and resuspended in 0.6 mL of loading Buffer A_scx_ (10 mM monopotassium phosphate (KH_2_PO_4_), 20% acetonitrile (MeCN), pH 3.0), followed by sonication. The pH was adjusted to 3.0 with 0.5 M orthophosphoric acid (H_3_PO_4_). The peptides were then separated by strong cation exchange chromatography as described previously (Fuller et al., 2015).

Each SCX fraction was analyzed by nanoLC ESI MSMS using a TripleTOF 5600 tandem mass spectrometer (ABSciex, Foster City, CA) as described previously (Fuller et al., 2015). The raw mass spectrometry data file was subsequently analyzed using ProteinPilot 4.5 software with the Paragon™ and ProGroup™ algorithms (ABSciex) against the human sequences in the Swiss-Prot database (http://www.uniprot.org/, accessed in July 2013; containing 539,616 sequences in total and 20,255 human sequences). Searches were performed using the preset iTRAQ settings in ProteinPilot. Trypsin was selected as the cleavage enzyme and MMTS modification of cysteines with a “Thorough ID” search effort. ProteinPilot's Bias correction, which assumes that most proteins do not change in expression and corrects for unequal mixing during the combining of labeled samples, was applied, with ratios of 1.0234 for the 115:114 labels, 0.6653 for 116:114, and 1.2362 for 117:114. Finally, detected proteins were reported with a Protein Threshold [Unused ProtScore (confidence)] >0.05 and used in the quantitative analysis if they were identified with three or more peptides with 95% confidence or above. *P*-values for the iTRAQ ratios were calculated by the ProteinPilot software. A False Discovery Rate (FDR) analysis was also performed against a concatenated database of forward and reverse protein sequences as a decoy database, using the Proteomics System Performance Evaluation Pipeline (PSPEP) in ProteinPilot, which reported 2093 proteins above a 5% local (for a given peptide) false discovery rate threshold and 2217 proteins above a 1% global false discovery rate threshold.

### Immunohisto/cytochemistry

**Figure 1:**
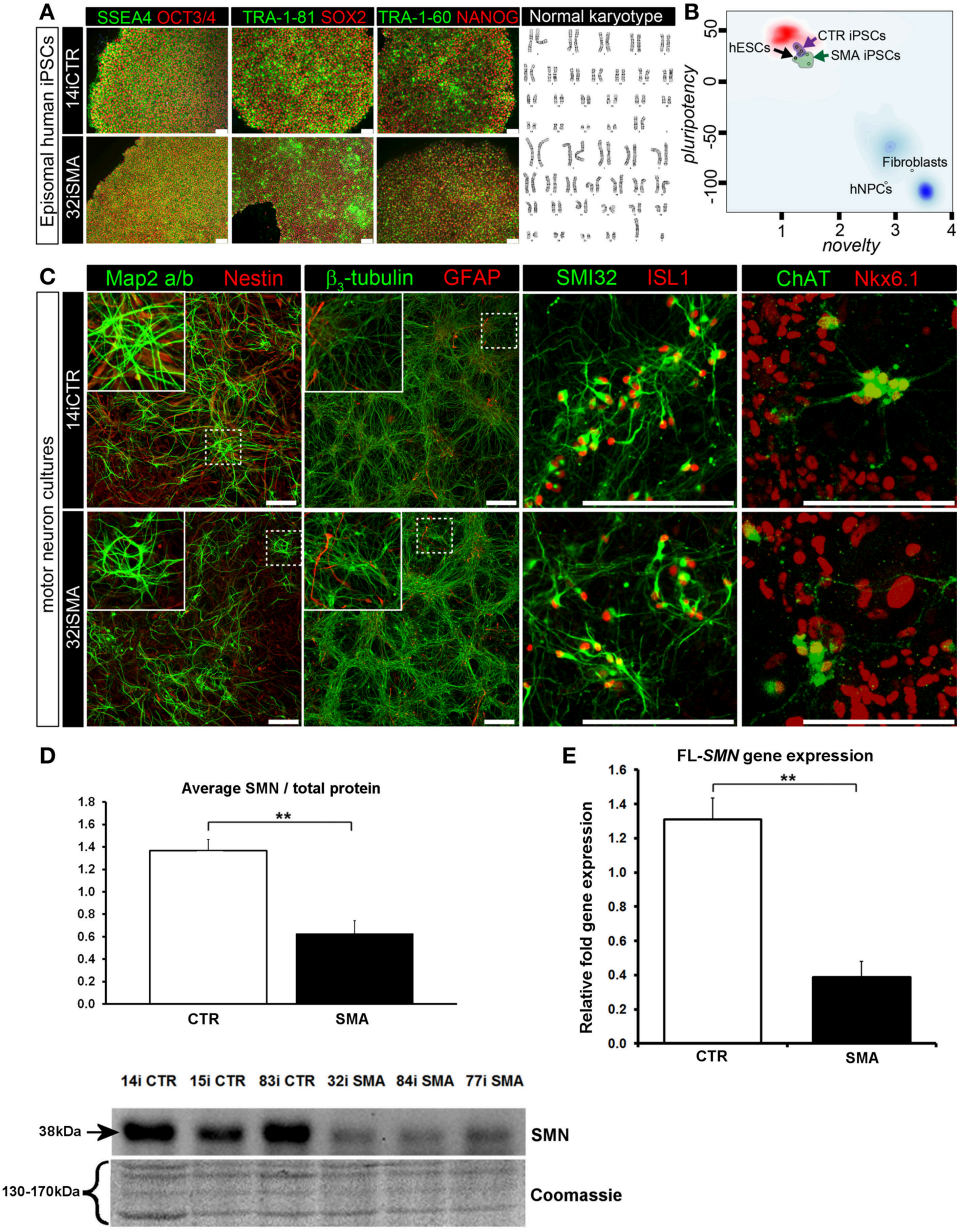
**Characterization of iPSC line and neuronal cultures representative of a healthy control and SMA Type 1 patient iPSC line. (A)** Representative positive immunostaining for nuclear and surface pluripotency antigens with normal G-band karyotype of the iPS cells shown at the right. **(B)** Gene-chip and bioinformatics based PluriTest characterization of control and SMA iPS cell lines used in this study. H9 human embryonic stem cells (hESCs) were used as positive control, while human dermal fibroblasts and primary human neural progenitor cells (hNPCs) were negative controls. **(C)** Upon neuronal induction and differentiation to the cultures analyzed contain: few Nestin progenitors (< 10%) and Map2 a/b neurons (dendritic marker), pan-neurons marker beta III-tubulin (>60%) with few astroglial (GFAP) cells, mostly SMI32- and ISL1 (Islet-1) positive motor neurons (~40%). Nkx6.1 and ChAT are spinal motor neuron markers that are expressed in both control and SMA-derived motor neurons. Scale bar for A is 75 μm. Scale bar for C is 200 μm. **(D)** Representative western blot showing SMN protein levels in three different control and SMA motor neuron cell lines, along with Coomassie stained gel as loading control. The graph represents mean integrated density of SMN bands from this blot/total protein (Coomassie gel), as determined by ImageJ software. Error bars represent standard error from the mean and statistical significance was calculated using an unpaired, one-tailed *t*-test with two-sample unequal variance. Please note that the Coomassie loading control shown here is the same that is shown for **Figure 4A** because they were both derived from the same blot. **(E)** Average gene expression levels of full-length (FL)-*SMN* in the control and SMA motor neurons [the same six cell lines shown in **(D)**], as determined by qRT-PCR (*p* = 0.002; unpaired, one-tailed *t*-test). Relative fold expression was normalized to H9 hESCs. Error bars represent standard error from the mean. ^**^p ≤ 0.01.

**Figure 2:**
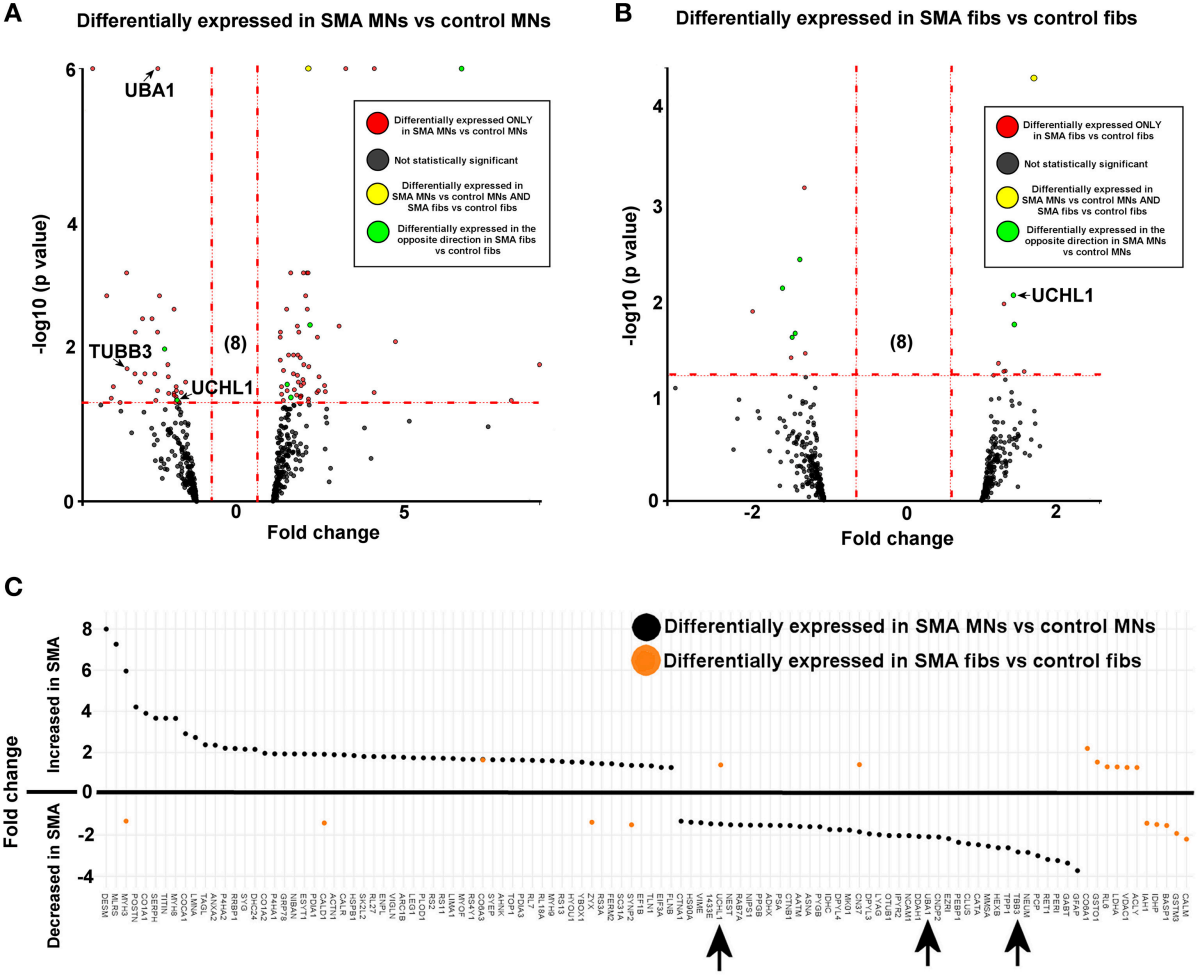
**SMN depletion has contrasting downstream effects on the proteome of motor neurons compared to genetically matched fibroblasts**. While 99 proteins were differentially expressed with statistical significance when SMA motor neurons (*n* = 1) were quantitatively compared to control motor neurons (*n* = 1) **(A)**, only 18 proteins were differentially expressed when SMA fibroblasts (*n* = 1) were quantitatively compared to control fibroblasts (*n* = 1) **(B)**. Of one these 18 proteins, only one was also differentially expressed in the same direction with statistical significance when the SMA MNs were compared to control MNs, and six of the 18 proteins were differentially expressed in one direction (i.e., up- or down-regulated) when SMA fibroblasts were compared to control fibroblasts and then expressed in the opposite direction when SMA MNs were compared to control MNs **(C)** (arrows indicate proteins that were verified biochemically). Plot **(C)** was generated using Plotly software (Plotly Technologies Inc. (2015), https://plot.ly).

**Figure 3:**
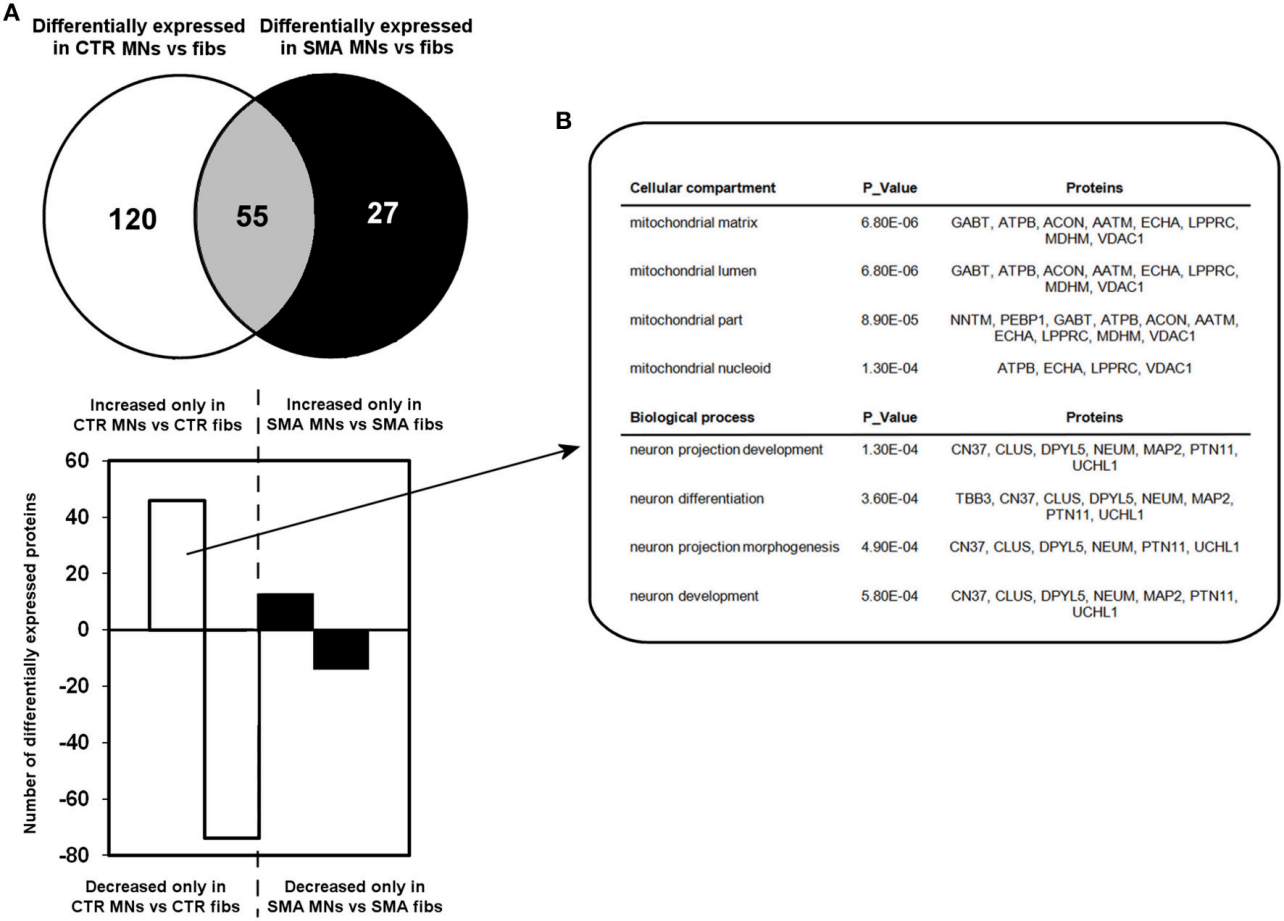
**Perturbation of developmental and differentiation pathways in SMA motor neurons. (A)** A Venn diagram and bar chart illustrates the number of differentially expressed proteins seen in control motor neurons (*n* = 1) compared to control fibroblasts (*n* = 1) (blue circle and blue bars) and SMN motor neurons (*n* = 1) compared SMA fibroblasts (*n* = 1) (green circle and green bars). **(B)** Bioinformatics analysis of the 46 proteins that were only increased in the control motor neurons vs. control fibs was conducted using the Database for Annotation, Visualization and Integrated Discovery (DAVID). CTR, control; MNs, motor neurons.

**Figure 4:**
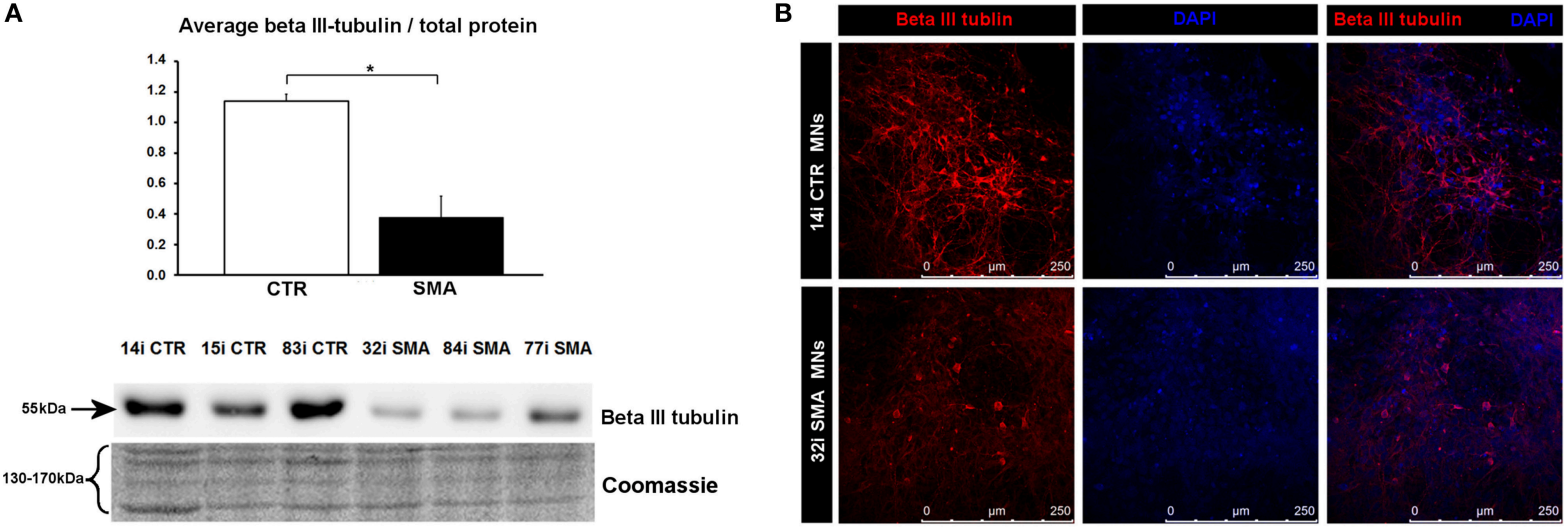
**Reduction of beta III-tubulin levels in SMA motor neurons. (A)** Representative western blot showing beta III-tubulin protein levels in three different control and SMA motor neuron cell lines, along with Coomassie stained gel as loading control. The graph above it illustrates the average integrated density of the beta III-tubulin bands from this blot/total protein (Coomassie gel), as determined by ImageJ software. Error bars represent standard error from the mean and statistical significance was calculated using an unpaired, ced *t*-test with two-sample unequal variance. Please note that the Coomassie loading control shown here is the same that is shown for Figure 1D because they were both derived from the same blot. **(B)** Representative confocal images indicate a reduction of beta III-tubulin levels in SMI-32 positive 32i SMA motor neurons. CTR, control; MNs, motor neurons. ^*^p ≤ 0.05.

**Figure 5:**
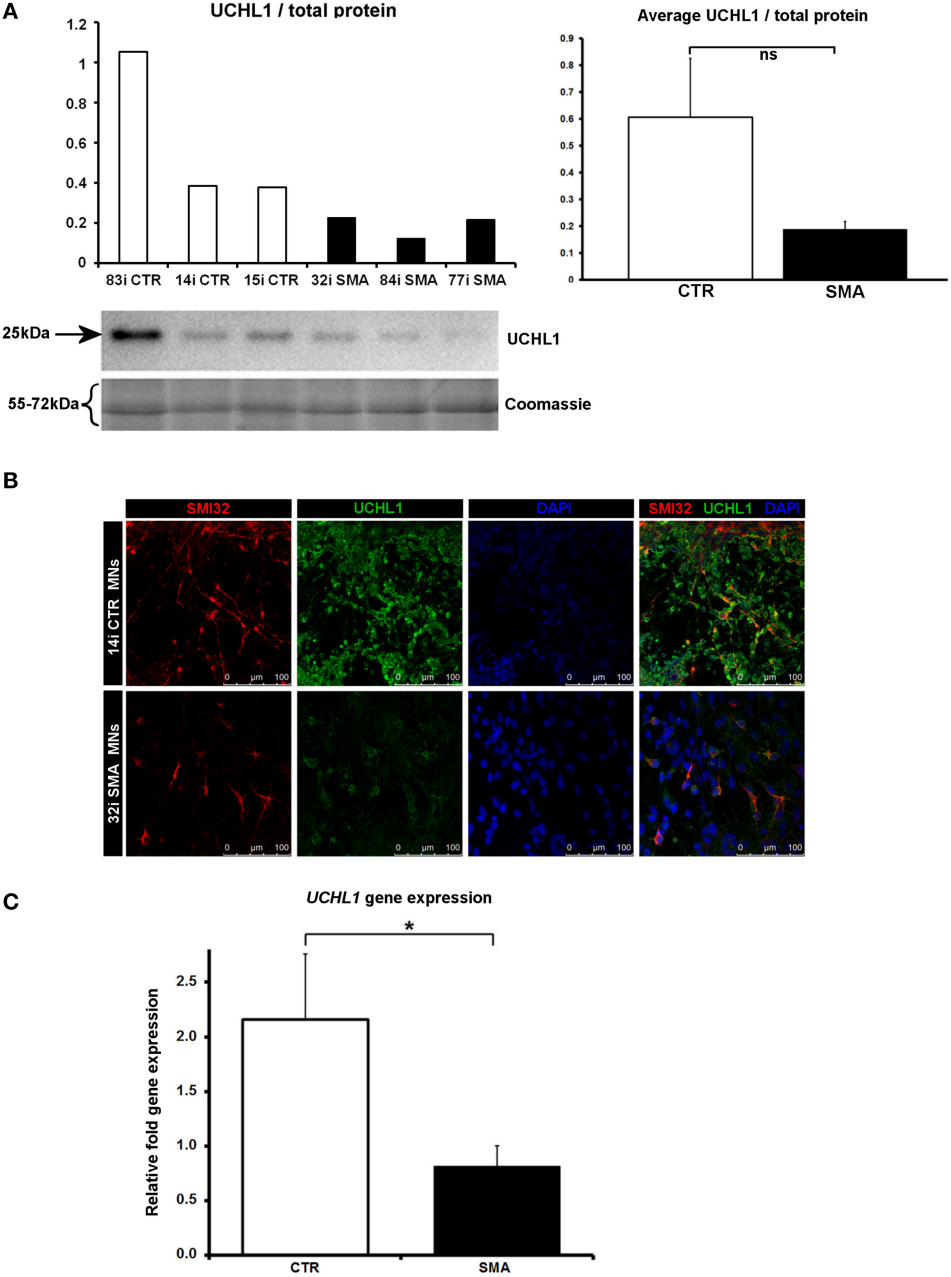
**Decreased UCHL1 levels in SMA motor neurons. (A)** Representative western blot showing UCHL1 protein levels in three different control and SMA motor neuron cell lines. The graph above it shows the integrated density of the UCHL1 bands from this blot/total protein (Coomassie gel), as determined by ImageJ software. The graph to the right shows the average integrated density of the UCHL1 bands from this blot/total protein (Coomassie gel). Error bars represent standard error from the mean and statistical significance was calculated using an unpaired, one-tailed *t*-test with two-sample unequal variance. **(B)** Representative confocal images indicate a reduction of UCHL1 levels in SMI32 positive cells. **(C)** Average gene expression levels of *UCHL1* in the control and SMA motor neuron cell lines [the same six cell lines shown in **(A)**], as determined by qRT-PCR (*p* = 0.04; unpaired, one-tailed *t*-test). Relative fold expression was normalized to H9 hESCs. Error bars represent standard error from the mean. CTR, control, MNs, motor neurons. ^*^p ≤ 0.05.

**Figure 6:**
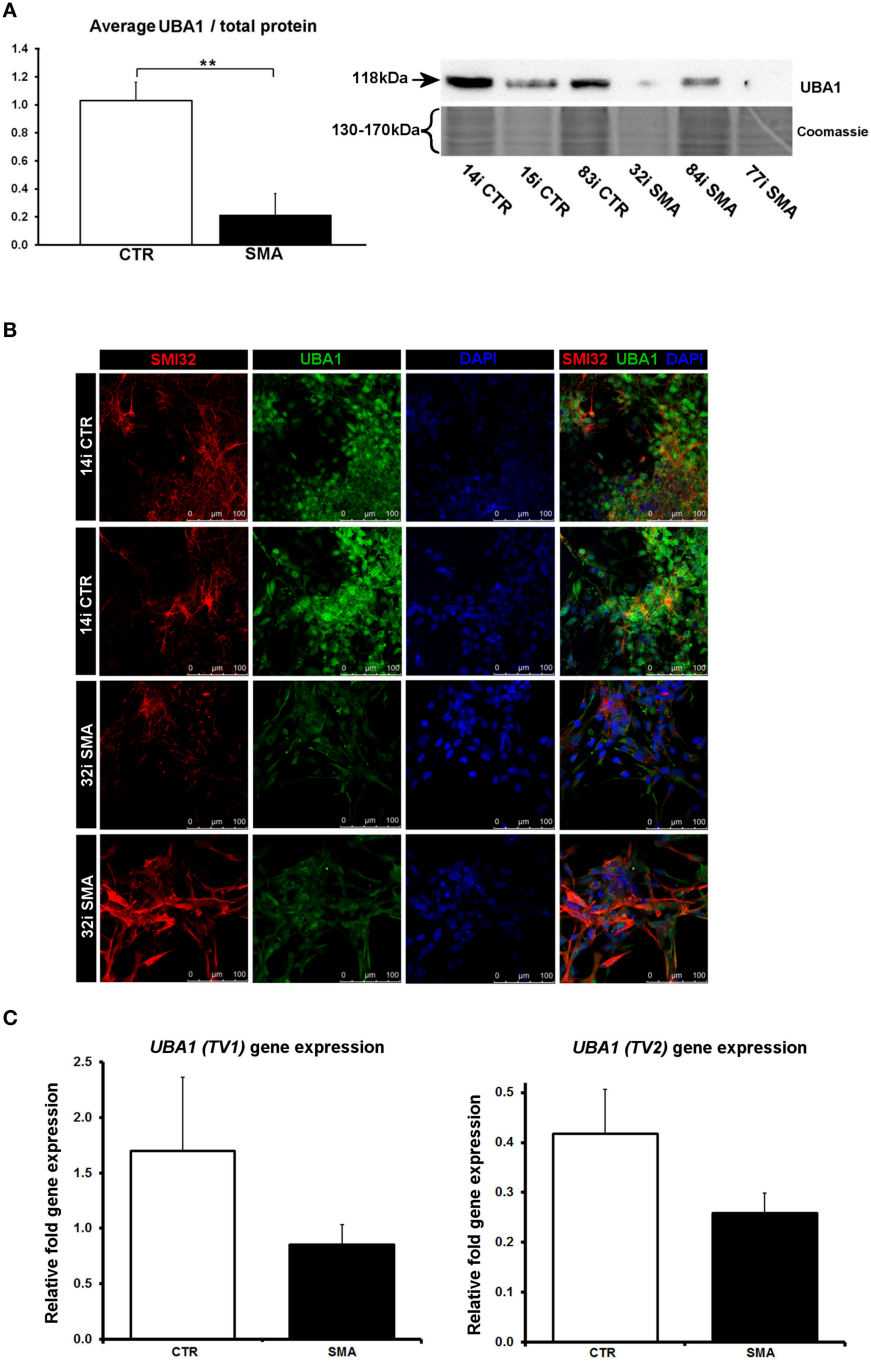
**Reduction and differential localization of UBA1 in SMA motor neurons. (A)** Representative western blot showing UBA1 protein levels in three different control and SMA motor neuron cell lines, along with Coomassie stained gel as loading control. The graph illustrates the average integrated density of the UBA1 bands from this blot/total protein (Coomassie gel), as determined by ImageJ software. Error bars represent standard error from the mean and statistical significance was calculated using an unpaired, one-tailed *t*-test with two-sample unequal variance. **(B)** Representative confocal images indicate a reduction of UBA1 levels in SMI32 positive 32i SMA motor neurons and mostly cytoplasmic distribution (in comparison to the mostly nuclear distribution seen in the 14i control (CTR) motor neurons). **(C)** Average gene expression levels of *UBA1* transcript variants 1 (TV1) (*p* = 0.14; unpaired, one-tailed *t*-test) and 2 (TV2) (*p* = 0.08; unpaired, one-tailed *t*-test) in the control and SMA motor neuron cell lines [the same six patient and control cell lines shown in **(A)**], as determined by qRT-PCR. Relative fold expression was normalized to H9 hESCs. Error bars represent standard error from the mean. CTR, control; MNs, motor neurons. ^**^p ≤ 0.01.

Human iPS cell lines and their differentiated cell types were plated on glass coverslips in optical-bottom 96-well plates (Thermo, # 165305) and subsequently fixed in 4% paraformaldehyde (Figure 1) or acetone/methanol (Figures 2–**6**). For Figure 1: cells were blocked in 5% normal donkey serum with 0.1% Triton X-100 and incubated with primary antibodies (Supplementary File S1) either for either 1 h at room temperature or overnight at 4°C. Cells were then rinsed and incubated in species-specific AF488, AF594, or AF647-conjugated secondary antibodies followed by Hoechst 33258 (0.5 μg/mL; Sigma) to counterstain nuclei. Cells were imaged using Molecular Devices Image Express Micro high-content imaging system or using Leica microscopes. For Figures 2–**6**: coverslips were incubated in primary antibodies (Supplementary File S1) for 1 h at room temperature. Cells were then rinsed and incubated for 1 h with 5 μg mL^−1^ goat anti-mouse ALEXA 488 (Molecular Probes, Eugene, OR) or swine anti-rabbit ALEXA 546, diluted in PBS containing 1% horse serum, 1% fetal bovine serum, and 0.1% BSA, followed by addition of DAPI (diamidinophenylindole 200 ng mL^−1^) for the final 5 min of incubation. After washing, coverslips were mounted on slides in Hydromount (Merck). High magnification images were obtained using a Leica SP5 confocal microscope with a 63X oil immersion objective.

### SDS-polyacrylamide gel electrophoresis and western blotting

Protein extracts from three separate SMA and three control iPSC-derived motor neuron cell lines were prepared by boiling in SDS sample loading buffer (2% SDS, 5% 2-mercaptoethanol, 62.5 mM Tris-HCl, pH 6.8), for 2 min. Proteins were subjected to SDS-PAGE (Biorad) using 12.5% polyacrylamide gels. A horizontal slice was excised from the gel (clear from the molecular weight of proteins of interest) for staining with Coomassie blue as an internal loading control. The proteins on the remaining part of the gel were then transferred to nitrocellulose membranes by western blotting. After blocking non-specific sites with 4% powdered milk solution, membranes were incubated with primary antibodies (Supplementary File S1), and diluted in dilution buffer (PBS, 1% fetal bovine serum, 1% horse serum, and 0.1% BSA). Antibody reacting bands were visualized by development with either peroxidase-labeled goat anti-mouse Ig or peroxidise-labeled swine anti-rabbit Ig (1 μg/mL in dilution buffer) and a chemiluminescent detection system (West Pico or West Femto, Pierce), followed by visualization using a Gel Image Documentation system (Biorad). Densitometry measurements of antibody reactive bands were derived using Image J software (v1.46) and were normalized to the densitometry of the Coomassie stained gel, as described by Eaton et al. (2013). For quantification of the Coomassie gel, a rectangular box was drawn around several bands in each lane for densitometry measurement (the details of the molecular weight range of these bands in each case is provided in each figure). The box was then copied and carefully pasted to the same position for every sample lane. In instances where the image quality of the Coomassie stained gel was low due to low protein levels (i.e., Figures 1D, **4**), the contrast was adjusted uniformly across the gel to enhance the signal. Unpaired, heteroscedastic *t*-tests were conducted (Microsoft Excel) to assess whether differences in densitometry were statistically significant.

### Quantitative RT-PCR

Total RNA was isolated from three separate SMA and three control iPSC-derived motor neuron cell lines using the Quick-RNA MiniPrep kit (Zymo Research). A volume of 2 μg of RNA was reverse transcribed using a High Capacity cDNA Reverse Transcription Kit by Applied Biosystems. Reactions were performed in triplicate using SYBR Select Master Mix (Applied Biosystems) using specific primer sequences (Supplementary File S3). Samples were held at 50°C for 2 min, 95°C for 2 min, and then cycled 40 times between 95°C for 15 s and 60°C for 30 s. A melting curve was recorded from 65 to 95°C in 0.5°C increments over 0.05 s steps. Expression of target genes was normalized to the expression of RPL13 ribosomal Protein L13A and calculated by the DDCT method (Livak and Schmittgen, 2001; Schmittgen and Livak, 2008). Unpaired, one-tailed *t*-tests were conducted using Graphpad Prism software, to assess the statistical significance of the expression data.

## Results

### Generation of human induced pluripotent stem cell-derived motor neurons

Induced pluripotent stem cells (iPSCs) generated from type I SMA patients and healthy controls (see Supplementary File S2 for origin, clinical history, and genetics) were generated as previously described (Barrett et al., 2014). Positive immunostaining confirmed the presence of nuclear and surface pluripotency antigens, along with normal G-band karyotype (Figure 1A). A gene-chip and bioinformatics based PluriTest (Müller et al., 2008–2012) characterization of the control and SMA iPS cell lines confirmed pluripotency in all SMA and control iPSC lines (determined by the presence of a PluriTest score of >20 in pluripotency and below 1.6 in novelty; Figure 1B). The red cloud and surrounding region signifies pluripotent samples, while anything outside the red cloud and in the blue area are non-pluripotent samples, based on well-characterized pluripotent stem cell data set (Müller et al., 2008–2012). The SMA and control iPSCs were then directed toward a lower spinal motor neuron fate by following stepwise differentiation paradigm mimicking human spinal cord development. The iPSCs first underwent neuroectodermal specification followed by addition of caudo-ventralizing factors (all-trans retinoic acid and sonic hedgehog agonist, purmorphamine) and maturation. At this point, the motor neurons are electrophysiologically active, as we have demonstrated previously (Sareen et al., 2013). These motor neuron cultures contained few nestin progenitors and Map2 a/b neurons (dendritic marker) (< 10%), pan-neurons marker βIII-tubulin (>60%) with few astroglial (GFAP) cells, and mostly SMI32 and ISL1 (Islet-1) positive motor neurons (~40%). Nkx6.1 and ChAT are spinal motor neuron markers that are expressed in both control and SMA-derived motor neurons (Figure 1C).

### SMN depletion has contrasting downstream effects on the proteome of motor neurons compared to genetically matched fibroblasts

To determine the downstream proteomic consequences of reduced SMN in iPSC-derived motor neuron cultures, we conducted a 4-plex quantitative comparison of the proteome of 32i SMA motor neurons with 14i control motor neurons, alongside the fibroblast cell lines from which they were originally derived (i.e., GM00232 and GM03814) using iTRAQ-mass spectrometry. This approach detected (and subsequently quantified) 2093 proteins, with a 5% local false discovery rate threshold (Supplementary Table 1). Even with the very latest technology, identification of proteins using mass spectrometry is limited to approximately the top 30% of the total proteome (Hornburg et al., 2014). It is possible, therefore, that due to the limitations of the technique, some important changes may not have been detected. For example, neither SMN nor any other known components of the SMN core complex were among the 2093 proteins that were detected and quantified, presumably because the SMN core complex appears to be a minor component of the entire cellular proteome (Fuller et al., 2010). Nonetheless, a statistically significant reduction of SMN protein in the SMA motor neurons was verified by western blotting (Figure 1D) and a reduction of full-length SMN gene expression was verified by RT-PCR (Figure 1E). A reduction of the SMN-binding protein, gemin2, was also seen by western blot analysis (Supplementary File S4), and is consistent with previous reports showing reduced levels of SMN complex components in SMA (e.g., Hao et al., 2007).

For reliable quantification, proteins identified from fewer than three peptides were excluded from the list, followed by exclusion of those with average iTRAQ ratios of < 1.25 or < 0.75, and finally exclusion of those with a *p* > 0.05. It is possible that the filtering criteria applied here may have resulted in some genuinely differentially expressed proteins being omitted from the final analysis, and so we have supplied supplementary tables of raw data to enable researchers to analyse the data differently, or to select other protein targets for further study (Supplementary Tables 1, 2). After applying the filtering criteria, 99 proteins were differentially expressed, with statistical significance, when SMA motor neurons were compared to control motor neurons (bold; Supplementary Table 1A and Figure 2A). The differential expression of several of the 99 proteins can be attributed to presence of some GFAP positive astrocytes, desmin-positive myoblasts in the original fibroblasts and collagen VI positive cells (Supplementary File S5). Although, an equal total protein concentration was loaded onto each iTRAQ tag, it is not yet possible to derive precisely synchronized, homogeneous populations of mature neurons from iPS cells.

When SMA fibroblasts were quantitatively compared to control fibroblasts, 18 proteins were differentially expressed (Supplementary Table 1B; bold font and Figure 2B). Interestingly, only one of these 18 proteins, collagen alpha-3 VI, was also differentially expressed in the same direction when the SMA motor neurons were compared to control motor neurons (*p* = 0.00004) (Supplementary Table 1 and Figure 2). Six of the 18 proteins were differentially expressed in one direction (i.e., up- or down-regulated) when SMA fibroblasts were compared to control fibroblasts and then expressed in the opposite direction when SMA MNs were compared to control MNs (Table 1, Supplementary Table 1 and Figure 2).

**Table 1:** **Differentially-expressed proteins with opposite expression levels in SMA fibroblasts compared to SMA motor neurons from the same patient**.

**Protein name**	**Accession number**	**Sequence coverage (%)**	**Avg. iTRAQ ratio (SMA fibs/CTR fibs)**	**Avg. iTRAQ ratio (SMA MNs/CTR iMNs)**
Myosin-3	P11055	52.5 [122]	0.75	5.95
Caldesmon	Q05682	42.0 [23]	0.71	1.92
Zyxin	Q15942	27.1 [18]	0.72	1.47
Synaptopodin-2	Q9UMS6	10.0 [3]	0.66	1.38
UCHL1	P09936	56.1 [37]	1.40	0.68
CNP	P09543	30.6 [3]	1.41	0.54

*Differentially-expressed proteins that were changed in one direction (i.e., up- or down-regulated) when SMA fibroblasts were compared to control fibroblasts, but changed in the opposite direction when SMA motor neurons were compared to control motor neurons. Only statistically significant differential expression (i.e., those with p < 0.05) are shown. Abbreviations in column headings refer to the following: protein name, given name in Swiss-Prot database; [n], number of unique peptides used for quantification; fibs, fibroblasts; SMA MNs, 32i motor neurons; CTR MNs, 14i control motor neurons*.

### Dysregulation of developmental and differentiation pathways in SMA motor neurons

A quantitative comparison of protein expression in the control motor neurons compared to their respective genetically matched fibroblast cells revealed that 175 proteins were differentially expressed, whereas just 82 proteins were differentially expressed in the SMA motor neurons when compared to the fibroblasts from which they were derived (Supplementary Tables 1C,D, 2A–C). Only 55 of these differentially expressed proteins were common to both the SMA and the control cells; of these, 17 proteins were increased in expression in the motor neurons compared to the fibroblast cells (Supplementary Tables 2A–C and Figure 3A).

To gain some insight into the likely functions of the 46 proteins that were increased in expression only in the control motor neurons vs. control fibs (and not increased in the SMA motor neurons vs. SMA fibs), gene ontology analysis was performed using the the Database for Annotation, Visualization and Integrated Discovery (DAVID; Huang et al., 2009a,b). Functional annotations that were assigned to fewer than three proteins and with a *p* > 0.05 were eliminated from the list. A clear enrichment of proteins of mitochondrial origin was detected, along with enriched biological process terms associated with neuronal development and differentiation (Figure 3B). The significant reduction of one such protein, beta III-tubulin (TBB3), to approximately one third of normal levels in three individual SMA motor neuron cell lines was verified by western blotting (Figure 4A) and is in very close agreement with the iTRAQ ratio (iTRAQ ratio 0.35; Supplementary Table 1A). Immunocytochemistry analysis revealed a global reduction of beta III-tubulin in both cell bodies and cellular processes of SMA motor neurons compared to control motor neurons at the same number of weeks of differentiation (Figure 4B). Whilst the control motor neurons expressed beta III-tubulin at levels of more than four times the amount of that seen in the control genetically matched fibroblasts (iTRAQ ratio of 4.36), beta III-tubulin levels were not significantly increased in the SMA motor neurons compared to their respective, genetically-matched, fibroblasts (iTRAQ ratio 1.13; not significant). This was despite the fact that motor neuron and pan-neuronal markers were clearly evident and similar in these cells (Figure 1). These findings correlate well with previous work showing a 1.3- to 1.4-fold reduction in the total number of processes in the SMA motor neurons at late stages of differentiation (i.e., 7–10 weeks) compared to control motor neurons (Sareen et al., 2012).

### Ubiquitin carboxyl-terminal esterase L1 is decreased in SMA motor neurons

Another protein that was implicated in neuronal development (Figure 3B) (that was increased only in control motor neurons vs. control fibs), and of some interest already to SMA, is ubiquitin carboxyl-terminal esterase L1 (UCHL1). UCHL1 was among the six proteins that were differentially expressed in one direction (i.e., up- or down-regulated) when SMA fibroblasts were compared to control fibroblasts and then expressed in the opposite direction when SMA motor neurons were compared to CTR motor neurons (Table 1). Levels of UCHL1 appear to be elevated in SMA patient fibroblasts and SMA mouse models (Hsu et al., 2010; Wishart et al., 2014). Contrary to this, we observed a reduction of UCHL1 levels in SMA motor neurons compared to the control motor neurons by iTRAQ (ratio 0.68; *p* = 0.049), despite the original SMA fibroblasts containing higher levels than the control fibroblasts (ratio 1.39; *p* = 0.007) (Supplementary Tables 1A,B). This observation was supported by western blot, immunocytochemistry and qPCR analysis. Western blot analysis of UCHL1 protein levels in three individual SMA motor neuron cells lines, compared to three control motor neuron cell lines showed a similar trend (Figure 5A) (ns, *p* = 0.10; presumably due to the variation between different cell lines). Immunocytochemistry analysis of the 32i SMA motor neurons also indicated lower levels of UCHL1, compared to the 14i control motor neurons (Figure 5B). In addition, a statistically significant reduction by ~65% (*p* = 0.04) of *UCHL1* gene expression was detected in the same three SMA motor neuron cells lines compared to the same control motor neuron cell lines (Figure 5C).

### Ubiquitin-activating enzyme 1 is reduced and differentially localized in SMA motor neurons

Several of the differentially expressed proteins (Supplementary Table 1A, Figure 3) have previously been reported as differentially expressed in SMA, further supporting the validity of these iPS cells as a model for SMA, as well as the overall approach employed in this study. Calreticulin—increased here by 1.89-fold in the SMA motor neurons compared to the control motor neurons (Supplementary Table 1A)—was previously shown to be increased in SMA mouse muscle, SMA fibroblasts and SMA patient muscle biopsies by ~1.5-fold, on average, although considerable variability between patients was noted (Mutsaers et al., 2013). Mutations in the ubiquitin-activating enzyme 1 (UBA1; previously UBE1) have been reported to cause infantile-onset X-linked SMA (SMAX2; Ramser et al., 2008; Dlamini et al., 2013) and the levels of UBA1 were reduced by ~50% in SMA mouse spinal cord (Huang et al., 2009b), more than 60% in skeletal muscle (Huang et al., 2009a) and by 50% in SMA mouse Schwann cells (Aghamaleky Sarvestany et al., 2014). In close agreement with this, we observed a 0.48-fold reduction of UBA1 in the SMA motor neurons compared to the control motor neurons by iTRAQ mass spectrometry (Supplementary Table 1A). This reduction was verified by western blot analysis of SMA (*n* = 3) and control (*n* = 3) motor neuron cell lines (Figure 6A), by immunocytochemistry in SMI32-positive cells (Figure 6B) and a similar trend was noted by gene-expression analysis of *UBA1* transcript variants 1 and 2 (in the same six cell lines used for western blotting) (Figure 6C). In the control motor neurons, the vast majority of UBA1 was localized to the nucleus and this was in contrast to the mainly cytoplasmic-distribution seen in the SMA motor neurons (Figure 6B), even in cells with relatively high levels of SMI32 expression (fourth row, Figure 6B). It seems likely that this represents a developmental abnormality in the SMA motor neurons, since nuclear accumulation of UBA1 appears to correlate with neuronal maturation (Smith-Thomas et al., 1994; Wishart et al., 2014).

## Discussion

In this study, human iPSC-derived motor neurons were used to identify motor neuron-specific down-stream effects of reduced SMN, not found in fibroblasts, which may help to explain the particular vulnerability of motor neurons in SMA. Here, we provide evidence that motor neurons from SMA patients display abnormalities in developmental and differentiation pathway proteins, and that many of these molecular differences are distinct from those seen in the genetically matched fibroblasts.

A new approach for gaining insight into the molecular pathways involved the dysfunction and demise of motor neurons in SMA has been made possible by the development of iPSC-derived motor neuron models from multiple SMA patients (Ebert and Svendsen, 2010; Chang et al., 2011; Corti et al., 2012; Sareen et al., 2012; Yoshida et al., 2015). In addition to displaying the obvious requirements of such a model (including reduced SMN protein and displaying the desired characterizations of diseased motor neurons Ebert and Svendsen, 2010; Sareen et al., 2012), we have been able to further validate the model via the identification, at the gene and protein level, of downstream consequences of reduced SMN that have been identified in other SMA model systems (e.g., UBA1, Wishart et al., 2014).

Though UBA1 is probably best known as an integral player in the ubiquitin protein-degradation pathway, this cascade also has important regulatory functions for the differentiation, development, and growth of neuronal cells (Hamilton and Zito, 2013; Saritas-Yildirim and Silva, 2014). In addition to reduced levels of UBA1, we observed differential distribution within the cells, whereby the majority of UBA1 was localized in the cytoplasm in SMA motor neurons, in contrast to the mainly nuclear distribution seen in control motor neurons. It seems likely that the differential distribution seen in the SMA motor neurons represents a developmental delay or abnormality in these cells since nuclear accumulation of UBA1 correlates with neuronal maturation and differentiation in chick embryos (Smith-Thomas et al., 1994) and mouse motor neurons (Wishart et al., 2014). Further support for this hypothesis comes from a clinical case report highlighting neurodevelopmental abnormalities in an infant with a type of SMA caused by a mutation in the *UBA1* gene (SMAX2; Dlamini et al., 2013). Wishart et al. (2014) reported that nuclear accumulation of UBA1 occurs in control and also SMA mouse motor neurons sometime between postnatal day 3 (P3) and 7, by which time, a reduction of cytoplasmic UBA1 staining intensity was noted in the SMA mice. It will be of interest, in the future, to determine whether the rate of subcellular redistribution of UBA1 differs between SMA and control mice, and in other cell and animal models.

Previous studies have highlighted neuro-developmental defects in SMA in primary tissue from zebrafish, mouse models, post-mortem patient spinal cord, and SMN-depleted germline stem cells from *Drosophila* (Simic et al., 2008; Murray et al., 2010; Grice and Liu, 2011; Hao le et al., 2015). However, many of these studies are limited in the fact that they were conducted using transgenic animal models or in late-symptomatic SMA patients. Little is known, therefore, about the precise molecular pathway(s) underlying the series of events that lead to these abnormalities *in vivo* and how well these models reflect the disease etiology seen early on in SMA patients. The expression of beta III-tubulin is well-known to be associated with differentiation and decreased cell proliferation of neurons (Katsetos et al., 2003). Neural stem cells (NSCs) from a mouse model of very severe SMA produce fewer beta III-tubulin-positive cells, and those that are produced, have fewer and shorter neurites; suggestive of neurodevelopmental abnormalities (Shafey et al., 2008). Such an observation, however, has never been confirmed in quantitative manner in SMA patient-derived neurons. Here we describe a global reduction of beta III-tubulin protein levels by iTRAQ, western blotting and immunocytochemistry in SMA motor neurons. Alongside the reduction of beta III-tubulin-positive cells, Shafey et al. (2008) also observed an increase in the numbers of proliferative NSCs and nestin-positive cells; implying that these cells had not differentiated as they ought to (Shafey et al., 2008).

The observation that UCHL1 and other proteins associated with neuronal differentiation and development were reduced in SMA motor neurons compared to control motor neurons supports the hypothesis that aberrations in early neurodevelopmental pathway proteins play a key role in SMA pathogenesis. Although, levels of UCHL1 are increased in SMA patient fibroblasts (Supplementary Table 1; Hsu et al., 2010) and in mouse models (Powis et al., 2014; Wishart et al., 2014), our results indicate that UCHL1 occurs at lower levels in SMA motor neurons compared to control motor neurons. Though this is the first such observation, it is not so surprising when we consider what we know already about the role of UCHL1 in neurons. In addition to an essential role maintaining the structure and function of the mouse neuromuscular junction (Chen et al., 2010), patients with a loss-of-function mutation in *UCHL1* demonstrate early-onset progressive neurodegeneration (Bilguvar et al., 2013), and more recently, UCHL1 was shown to have a role in maintaining the viability of corticospinal motor neurons (Jara et al., 2015). Moreover, pharmacological inhibition of UCHL1 appears to exacerbate disease symptoms in a mouse model of SMA, suggesting that the increased levels seen in certain cell types may either be due to an attempted compensatory response or that these cells respond differently to reduced SMN, compared to motor neurons (Wiese et al., 2004). This finding also highlights the potential complexities of therapies for SMA aimed at restoring ubiquitin homeostasis (Wishart et al., 2014; Powis et al., 2014).

In a recent article by Hornburg et al. (2014), proteomics analysis was used to delineate differences between primary and cell line mouse models of motor neuron disease. The study placed neuronal cell line models halfway between primary motor neurons and unrelated cell lines, in terms of the proteomic-profile of the cells. Although, it is not possible to directly compare the proteome of primary human motor neurons with iPSC-derived motor neurons in a similar fashion, our results suggest that the motor neurons used in this study are far removed from their genetically matched fibroblasts, at least in terms of their proteomic profile. When SMA motor neurons were compared to control motor neurons, all but one of the 99 differentially expressed proteins were distinct from the differences seen when genetically matched fibroblast cell lines were compared. The differential expression of several of these candidates that have been implicated in SMA (i.e., SMN, beta III-tubulin and UBA1) have been verified here in three separate control and SMA iPS cell lines. Our results validate the potential of iPSC technology in identifying relevant disease mechanisms in motor neuron diseases by a proteomics approach and builds upon iPSC disease modeling studies that have been limited by number of independent (non-clonal) diseased patient cell lines (Bilican et al., 2012; Corti et al., 2012; Donnelly et al., 2013; Chen et al., 2014; Shan et al., 2014; Boza-Morán et al., 2015). In the future, it will be interesting to examine this further by label-free quantitative analysis of a larger cohort of patient-derived samples and to characterize the molecular implications of genetic diversity/heterogeneity of the cell lines.

Despite recent advances in our understanding of the molecular pathways involved in SMA (d'Ydewalle and Sumner, 2015), it is still not clear why lower motor neurons, in particular, are so vulnerable to reduced levels of SMN protein. Though previous longitudinal analyses suggests that both SMA and control iPS-derived motor neuron cultures undergo neurogenesis at similar levels over time (Sareen et al., 2012), it is conceivable that some proteomic differences identified here could reflect a delay in development that may not be apparent at a later stage, if the cells were able to “catch up” developmentally. It is important to consider, however, that a delay in any aspect of motor neuron development could itself be a fundamental difference with catastrophic consequences for motor neuron circuitry development. As such, if any proteomic changes are due to a slower development speed, they are still likely to be highly relevant to SMA. This notion is supported by the fact that early postnatal delivery of AAV9-SMN rescues the SMA mouse, while later delivery does not (Foust et al., 2010), indicating that there is a critical developmental time window.

The distinctly different patterns of differential expression seen here between SMA and control motor neurons, compared to the differential expression seen in genetically matched fibroblasts, indicates that SMN depletion has very different downstream consequences in different cell types. The proteomic differences identified here, therefore, are likely to provide a useful resource for exploring the molecular consequences of reduced SMN in motor neurons and for the identification of novel therapeutic targets for SMA. In particular, the robust finding here, of depleted levels of UBA1 in iPSC-derived motor neurons from three separate SMA patients compared to controls, supports a growing body of evidence (Ramser et al., 2008; Dlamini et al., 2013; Wishart et al., 2014)to suggest that this protein is likely to be a major contributor to pathogenesis in SMA.

## Author contributions

HF participated in the study design, conducted experiments, analyzed data, and wrote the manuscript. SS and CB conducted the mass spectrometry analysis. BM performed differentiation and culturing of the iPS cells. AG performed the qRT-PCR experiments. AK performed the western blot experiment in the Supplementary File. GM participated in the study design and provided reagents for the study. DS wrote manuscript, participated in the study design, conducted iPS cell characterization, neuronal differentiation experiments, and analyzed data. The manuscript was written through contributions of all authors. All authors have given approval to the final version of the manuscript.

### Conflict of interest statement

The authors declare that the research was conducted in the absence of any commercial or financial relationships that could be construed as a potential conflict of interest.
