# Supplementary material for: Spinal Muscular Atrophy Patient iPSC-Derived Motor Neurons Have Reduced Expression of Proteins Important in Neuronal Development
Source: Front Cell Neurosci. 2016 Jan 11;9:506. doi: 10.3389/fncel.2015.00506 (PMC4707261; doi:10.3389/fncel.2015.00506)
Supplement: Supplementary file 3 [file DataSheet1.DOCX]

**Spinal muscular atrophy patient iPSC-derived motor neurons have reduced expression of proteins important in neuronal development**

*Heidi R. Fuller^1,2^, Berhan Mandefro^3,4^, Sally L. Shirran^6^, Andrew R. Gross^3^†, Anjoscha Kaus^3^†, Catherine H. Botting^6^, Glenn E. Morris^1,2^, Dhruv Sareen^3,4,5*^.*

**Supplementary File**

**S1: Antibodies used for immunocytochemistry and immunoblotting.**

| Antigen | Dilution | Catalog # | Isotype | Manufacturer |
| --- | --- | --- | --- | --- |
| **SSEA4** | 1:250 | MAB4304 | mIgG3 | Millipore |
| **TRA-1-60** | 1:250 | 09-0010 | mIgM,κ | Stemgent |
| **TRA-1-81** | 1:250 | 09-0011 | mIgM,κ | Stemgent |
| **OCT4** | 1:250 | 09-0023 | Rabbit IgG | Stemgent |
| **NANOG** | 1:250 | 09-0020 | Rabbit IgG | Stemgent |
| **SOX2** | 1:500 | AB5603 | Rabbit IgG | Millipore |
| **TuJ1 (β_3_III-tubulin) (Figure 1)** | 1:1000 | T8535 | mIgG2b | Sigma |
| **TuJ1 (βIII-tubulin)**  **All other figures** | 1:500 | MMS-435P | Mouse IgG2a | Biolegend |
| **NKX6.1** | 1:100 | F55A10 | mIgG1 | DSHB Iowa |
| **ISELT1** | 1:250 | AF1837 | Goat IgG | R & D systems |
| **SMI32** | 1:1000 | SMI-32R | mIgG1 | Covance |
| **CHAT** | 1:250 | AB144P | Goat IgG | Millipore |
| **Map2 a/b** | 1:550 |  | Mouse IgG | SIGMA |
| **UCHL1** | 1:500 (WB), 1:100 (IF) | NB300-676 | Rabbit IgG | Novus Biologicals |
| **UBE1** | 1:5000 (WB),  1:50 (IF) | Ab133479 | Rabbit monoclonal IgG | Abcam |
| **SMI32** | 1:1000 | SMI-32R | Mouse IgG1 | Biolegend |
| **SMN** | 1:100 | MANSMA12 | Mouse IgG | Wolfson CIND |
| **GFAP** | 1:500 | Z0334 | Rabbit Ig | DAKO |
| **COL6A3** | 1:50 |  | Mouse | Gift from Prof C. Sewry |
| **Desmin** | 1:25 |  | Rabbit | Gift from Prof C. Sewry |
| **Gemin2** | 1:100 | MANSIP1A | Mouse IgG | Wolfson CIND |

**S2: origin, clinical history and genetics of each cell line.**

| iPS cell line | Parent tissue | Coriell identifier | Clinical history and genetics | Sex |
| --- | --- | --- | --- | --- |
| 14iCTR | Fibroblast | GM03814 | Clinically normal; 2 affected children; donor subject has two copies of the SMN2 gene; donor is heterozygous for deletion of exons 7 and 8 in the SMN1 gene | Female |
| 15iCTR | Fibroblast | GM03815 | Clinically normal; 2 affected children | Male |
| 83iCTR | Fibroblast | GM02183 | Clinically Normal; At risk (50%) for Huntington's Disease, 33 CAG repeats | Female |
| 32iSMA | Fibroblast | GM00232 | SMA Type I; donor subject has only one copy of the SMN2 gene and is homozygous for deletion of exons 7 and 8 of the SMN1 gene. | Female |
| 77iSMA | Fibroblast | [GM09677](https://catalog.coriell.org/0/Sections/Search/Sample_Detail.aspx?Ref=GM09677&Product=CC) | SMA Type I; Expired at age 23 months; negative family history; donor subject has 2 copies of the SMN2 gene and is homozygous for deletion of exons 7 and 8 of the SMN1 gene. | Male |
| 84iSMA | EBV B-Cells | [GM10684](https://catalog.coriell.org/0/Sections/Search/Sample_Detail.aspx?Ref=GM10684&Product=CC) | SMA Type I; donor subject is homozygous for deletion of exons 7 and 8 of the SMN1 gene and has 2 copies of the SMN2 gene. Negative family history. | Female |

**S3: qRT-PCR primers.**

| **Gene** | **Accession No.** | **Forward** | **Reverse** |
| --- | --- | --- | --- |
| **UBA1 (TV1)** | [NM_003334.3](http://www.ncbi.nlm.nih.gov/entrez/viewer.fcgi?db=nucleotide&id=163659922) | GGAACCGGCATTGATGTCCA | GCAGGGGAGCAGTTAGAACC |
| **UBA1 (TV1)** | [NM_153280.2](http://www.ncbi.nlm.nih.gov/entrez/viewer.fcgi?db=nucleotide&id=163659921) | TGTACGACAGAGGTGGTTTGC | GCGAGCTGGACATCCCCTG |
| **UCHL1** | [NM_004181.4](http://www.ncbi.nlm.nih.gov/entrez/viewer.fcgi?db=nucleotide&id=239835751) | GCTCCGCTAGCTGTTTTTCG | GTTCAGCATCTCGGGGTTGA |
| **SMN** | [NM_000344.3](http://www.ncbi.nlm.nih.gov/entrez/viewer.fcgi?db=nucleotide&id=196115055)  [NM_022874.2](http://www.ncbi.nlm.nih.gov/entrez/viewer.fcgi?db=nucleotide&id=196115040)  [NM_017411.3](http://www.ncbi.nlm.nih.gov/entrez/viewer.fcgi?db=nucleotide&id=196115210)  [NM_022876.2](http://www.ncbi.nlm.nih.gov/entrez/viewer.fcgi?db=nucleotide&id=196115223) | CAAAAAGAAGGAAGGTGCTCA | GTGTCATTTAGTGCTGCTCTA |
| **RPL13A** | [NM_012423.3](http://www.ncbi.nlm.nih.gov/entrez/viewer.fcgi?db=nucleotide&id=395132448)  [NM_001270491.1](http://www.ncbi.nlm.nih.gov/entrez/viewer.fcgi?db=nucleotide&id=395132449) | CCTGGAGGAGAAGAGGAAAGA | TTGAGGACCTCTGTGTATTTG |

**S4. Quantitative western blot showing a reduction of gemin2 in SMA motor neurons.**

**
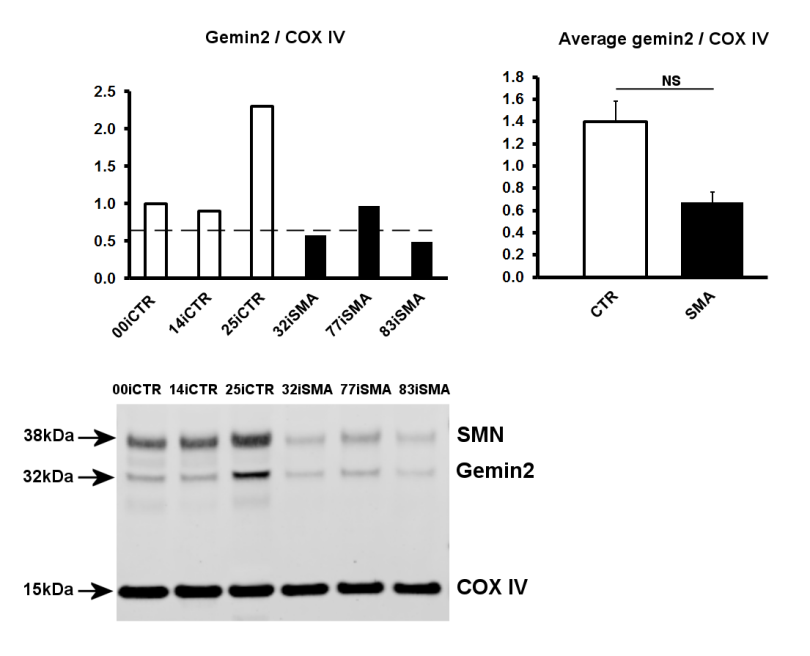
**

Western blot showing Gemin2, SMN and COX IV protein levels in three different control and SMA motor neuron cell lines. The graph above it shows the integrated density of the gemin2 bands from this blot / COX IV (loading control). The dotted line indicates the average integrated density of gemin2 / COXIV across the three SMA motor neuron cell lines. The graph to the right shows the average integrated density of the gemin2 bands from this blot / COX IV (loading control). Error bars represent standard error from the mean and statistical significance was calculated using an unpaired, 1-tailed t-test with two-sample unequal variance.

**
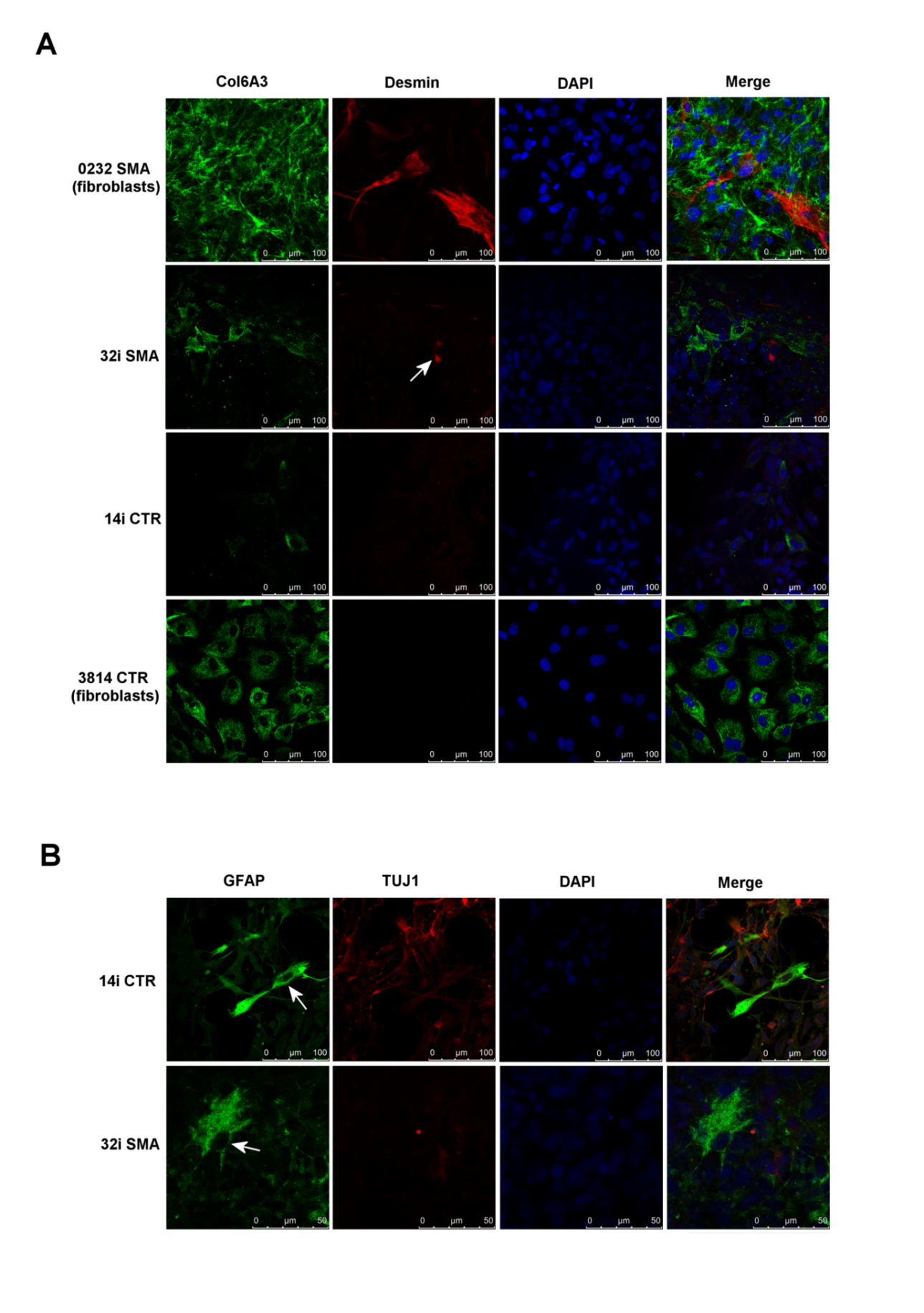
S5. Immunofluorescence staining of iPS-derived motor neurons and fibroblasts.**

(A) Desmin positive cells (red) were detected in 0232 fibroblasts and 32i SMA motor neurons. Collagen 6, alpha 3 (COL6A3) (green) positive cells were detected both fibroblasts lines and iMN cultures, though 32i SMA cells appeared to have a greater % of COL6A3 cells compared to the 14i control (CTR) cells. (B) Type 1 astrocytes were detected in both iMN cell lines (green).
